# LIVING WITH DEMENTIA: AN INTERDISCIPLINARY COMMUNITY-BASED EDUCATION SERIES TO EMPOWER CARE PARTNERS

**DOI:** 10.1093/geroni/igad104.1658

**Published:** 2023-12-21

**Authors:** Erica Husser, Marie Boltz

## Abstract

Approximately 75% of persons living with dementia receive essential day-to-day direct care and support from informal, unpaid care partners who receive little education or guidance from health care professionals on how to negotiate day-to-day challenges associated with living with dementia. This symposium describes the development, implementation, and impact of a comprehensive, four-part Living with Dementia community education program focused on dementia care featuring an optional Memory Café, developed collaboratively by the Penn State College of Medicine and College of Nursing for the purpose of empowering care partners. The first presentation will describe the educational content and instructional strategies, designed by an interdisciplinary team (physician, nurse, and long-term care dementia specialist), and based on the Medicare Cognitive Assessment and Care Plan Services visit and using evidence-based curricula. The second presentation describes the concurrent and collocated intergenerational respite program (Memory Café) for persons living with dementia (PLWD) that enabled care partners to attend the education. Health care students were paired with a PLWD and during the two-hour session each pair engaged in Opening Minds though Art™ (OMA), reminiscence, and music therapy; outcome data includes PLWD pre- and post-mood assessments. The third presentation provides overall evaluation data from the 4-part education series. Respondents were asked about the overall impacts of the program, the skills they learned, the usefulness of the education now and into the future, and what additional support would be most helpful. Our discussant will address the implications of our findings for practice, policy, and future program development.

